# Match Analysis in Team Ball Sports: An Umbrella Review of Systematic Reviews and Meta-Analyses

**DOI:** 10.1186/s40798-022-00454-7

**Published:** 2022-05-13

**Authors:** Hugo Sarmento, Filipe Manuel Clemente, José Afonso, Duarte Araújo, Miguel Fachada, Paulo Nobre, Keith Davids

**Affiliations:** 1 University of Coimbra, Research Unit for Sport and Physical Activity (CIDAF), Faculty of Sport Sciences and Physical Education, Coimbra, Portugal; 2 Escola Superior Desporto e Lazer, Instituto Politécnico de Viana do Castelo, Rua Escola Industrial e Comercial de Nun’Álvares, 4900-347 Viana do Castelo, Portugal; 3 Center for Research, Education, Innovation and Intervention in Sport, Faculty of Sport, University of Porto, 4200-450 Porto, Portugal; 4 CIPER, Faculdade de Motricidade Humana, Universidade de Lisboa, Cruz Quebrada, Portugal; 5 Sport and Human Performance Research Group, Sheffield Hallam University, Sheffield, UK

**Keywords:** Team sports, Group behaviours, Ecological dynamics, Performance analysis

## Abstract

**Background:**

Match analysis has evolved exponentially over the past decades in team sports resulting in a significant number of published systematic reviews and meta-analyses. An umbrella review of the available literature is needed to provide an integrated overview of current knowledge and contribute to more robust theoretical explanations of team performance.

**Methods:**

The Web of Science (all databases), PubMed, Cochrane Library (Cochrane Database of Systematic Reviews), Scopus, and SPORTDiscus databases were searched for relevant publications prior to 19 February 2021. Appraisal of the methodological quality of included articles was undertaken using the tool for Assessing the Methodological Quality of Systematic Reviews (AMSTAR-2). Twenty-four studies were reviewed that met the following criteria: (1) contained relevant data from match analyses in team ball sports; (2) were defined as systematic reviews or/and meta-analyses; and (3) were written in the English language.

**Results:**

The overall methodological quality of the 24 included reviews, obtained through the AMSTAR-2, revealed very low confidence ratings (Critically Low, *n* = 12) for the results of most systematic reviews of match analyses in team ball sports. Additionally, the results showed that research is focused mainly on four levels of analysis: (1) dyadic (microlevel); (2) individual (molecular level; predominant); (3) group (mesolevel), and (4) team dynamics (macrolevel). These levels of analysis included tactical, technical, physical, and psychosocial variables. Team performance was contextualized at two levels, with reference to: (1) match context (e.g. match status, match location, match period, quality of opposition) and (2) sociodemographic and environmental constraints (sex, age groups, competitive level, altitude, temperature, pitch surface).

**Conclusions:**

The evolution of methods for match analysis in team ball sports indicates that: (1) an individual-level performance analysis was predominant; (2) the focus on intermediate levels of analysis, observing performance in dyadic and group interactions, has received less attention from researchers; (3) neglected areas of research include psychosocial aspects of team sports and women’s performance; and (4) analyses of match contexts need greater depth.

*Registration*: The protocol was registered in the International Platform of Registered Systematic Review and Meta-Analysis Protocols with the number 202080067 and the DOI number https://doi.org/10.37766/inplasy2020.8.0067.

**Supplementary Information:**

The online version contains supplementary material available at 10.1186/s40798-022-00454-7.

## Key Points

1. Although previous reviews have focused on team sports, player-level analyses of team performance remain dominant. The majority of reviews, which focus on the tactical dimension of performance, have reported outcomes related to group dynamics.
2. Future studies need to adopt more integrated and holistic analyses of team sports performance (i.e. by considering physical, technical, tactical, and psychosocial aspects) to bridge the gap between theory and practice.
3. Finally, research on how these four different levels of performance might interact is needed; such work requires a transdisciplinary framework, such as ecological dynamics, to interpret outcomes.

## Introduction

The impact of and interest in performance analysis in team sports, as a methodological approach to promote athlete development and preparation, have increased over the years. The first significant insights in this area of sports science were probably provided by Evers and Fullerton [1], who published “Touching Second: The Science of Baseball”. They developed their own ideographic notation system and demonstrated the early performance notation of baseball in the late nineteenth century. Nevertheless, scientific studies, including performance analysis, remained dormant until match analysis in team ball sports started to draw considerable attention in the 1980s and 1990s. Increasing scientific and technical research on match analysis has coincided with the creation of international scientific societies and specialized scientific journals, the constitution of autonomous research departments in higher education institutions worldwide, and the organization of scientific events meetings dedicated to enhancing methodological and statistical knowledge in this subdiscipline [2].

Performance analysis has evolved from providing statistics-based activity profiles of players during competitive matches to analyses of the frequency counts of technical actions [3]. Recently, researchers have focused on analysing tactical behaviours (i.e. individual or group, goal-directed, behavioural combinations) in various competitive contexts [3]. This body of work has constituted a significant investigation of team ball sports focusing on the performance of actions during games. Such work is intended to detect regular structures (patterns of play) with different contextual constraints applied (see González-Víllora et al. [4] for information concerning different tactical analysis tools in team sports). Additionally, the proliferation of technological systems (e.g. global positioning system [GPS], Prozone—STATS, OPTA) to collect performance data has led to an exponential development in this area of research [5] and an increased sophistication of data analysis techniques.

Understanding how individual and subgroup or team behaviours emerge during interactions represents a fundamental issue for performance analytics in sports sciences [6]. Match analysis is intended to provide a theoretical understanding of collective and individual human behaviours as they emerge under varying constraints within competitive performance contexts. For example, Araújo and Bourbousson [6] discussed the strengths, weaknesses, and implications of three theoretical perspectives (social-cognitive, enactive, and ecological dynamics) on coordination in group behaviours. Considering the extraordinary complexity of human group behaviours and how little is known about them, they argued for explanatory pluralism in (social) psychology. In other words, they proposed that theoretically informed research would generate more precise definitions of specific hypotheses. They also discussed the need to clarify the intricacies of group behaviours in terms of emergent dynamics, intentionality, coordination, and adaptation. For example, the systems-oriented theory of synergy formation in teams [7] indicates that four properties are linked to successful performance: dimensional compression, reciprocal compensation, division of labour or interpersonal linkages, and system degeneracy. Research considering how these properties should be recorded, seeking other properties, and exploring how they interact with each other would enhance explanations for group behaviour.

Moreover, theoretically informed performance analysis research could explain the connections between the individuals, subgroups, and the entire social system developed in team sports competition [8]. For example, some theories of team synergy formation focus on the interactive, systemic relationship that emerges between each individual and a performance environment. In this way, such theories aim to explain how each individual contributes to group and team behaviours as captured by system properties such as *division of labour*. In contrast, research on performance analysis tends to examine the role of technological advancements, thus facilitating inductive research. This claim is exemplified by the emphasis on big data and artificial intelligence [9] in sports performance, as data are mined to reveal hidden patterns and structures. However, such inductive approaches would benefit from a link with theoretically informed approaches to collective human behaviours to investigate the performance–environment relationship [10].

Many empirical studies and systematic reviews have been published in this scientific area, and the number of such publications has increased exponentially in the last few years. Therefore, it is important to undertake umbrella reviews to (1) inform researchers about the evolution of knowledge on performance analysis across different team ball sports; (2) describe new techniques for gathering information about team ball play; and (3) provide a theoretical conceptualization of key findings and observations to continue to update a conceptual basis for performance analysis in team ball sports [11, 12].

The aim of the present paper is threefold: (1) to systematically examine available systematic reviews and meta-analyses on match analysis in team ball sports; (2) to evaluate the quality, strengths, and limitations of peer-reviewed, published evidence; and (3) to identify current gaps in the literature that can be addressed in future research.

## Methods

An umbrella review of systematic reviews and meta-analyses was conducted according to Preferred Reporting Items for Systematic Reviews and Meta-analysis (PRISMA) guidelines [13, 14].

### Registration and Protocol

The protocol was registered in the International Platform of Registered Systematic Review and Meta-Analysis Protocols with the number 202080067 and the DOI number https://doi.org/10.37766/inplasy2020.8.0067.

### Search Strategy: Databases and Inclusion Criteria

In the first phase of a systematic process, the Web of Science (all databases), PubMed, Cochrane library (Cochrane Database of Systematic Reviews), Scopus and SPORTDiscus databases were searched for relevant publications prior to 19 February 2021. The following search terms were included in Boolean search strategies: (“match analysis” OR “performance analysis” OR “notational analysis” OR “game analysis” OR “tactical analysis” OR “patterns of play”) AND (“team sport*” OR football OR soccer OR futsal OR handball OR volleyball OR basketball OR hockey OR rugby OR cricket OR “water polo” OR lacrosse OR softball OR korfball) AND (review OR meta-analysis). Next, reference lists in the studies recovered were hand searched to identify potentially eligible studies not captured by electronic searches. Finally, an external expert was contacted to verify the final list of references included in the umbrella review to identify specific studies that were not detected through our research.

The publications included in the first search round met the following predefined inclusion and exclusion criteria: (1) contained relevant data concerning match analysis in male and female team ball sports; (2) were systematic reviews or/and meta-analyses; and (3) were written in the English language. Studies were excluded if they (1) included data from other sports; (2) did not contain any relevant data on match analysis in team ball sports; (3) were written in a language other than English; and (4) were empirical studies or narrative reviews. The option for the English language was included because major, established journals are all published in English.

Two reviewers (HS and FC) independently screened the title, abstract, and reference list of each study to locate potentially relevant studies and reviewed the full version of the included papers in detail to identify articles that met the selection criteria. A third external reviewer (JA) was consulted to resolve any discrepancies regarding the selection process.

### Quality of the Studies and Extraction of Data

The methodological quality of the included articles was evaluated using the Assessing the Methodological Quality of Systematic Reviews (AMSTAR-2) tool [15], which consists of 16 items. The quality of each eligible article was independently analysed by two researchers (HS, FC). Both researchers had experience in conducting systematic reviews and meta-analyses and using AMSTAR-2. Whenever a disagreement arose between the two researchers’ evaluations, a consensus was reached either by discussion or with the help of a third reviewer (see Additional file 1: Table S1). Interrater (Kappa) agreement ranged from 0.53 (weak) to 1.00 (almost perfect), as suggested by McHugh [16].

Overall confidence in the results of each systematic reviews was defined by the AMSTAR-2 tool and expressed as follows: (1) “High”—zero or one noncritical weakness: the systematic review provides an accurate and comprehensive summary of the results; (2) “Moderate”—more than one noncritical weakness but no critical flaws: the systematic review provides an accurate summary of the results; (3) “Low”—one critical flaw, with or without noncritical weaknesses: the systematic review may not provide an accurate and comprehensive summary of the results; or (4) “Critically low”—more than one critical flaw, with or without noncritical weaknesses: the review should not be relied on to provide an accurate and comprehensive summary of the results. The original critical domains that can critically affect the validity of a review and its conclusions, as proposed by Shea and Reeves [15], were considered in this review.

Data were extracted by two investigators (FC and JA) and checked by a third investigator (HS). A template for data extraction was developed. For each included study, the following items were extracted: citation details, purpose of the study and context of analysis, type of analysis, match context, individual and environmental constraints, and main study outcomes. Because the data were descriptively reported in this study, no statistical analyses were undertaken.

## Results

### Search, Selection, and Inclusion of Publications

**Fig. 1:**
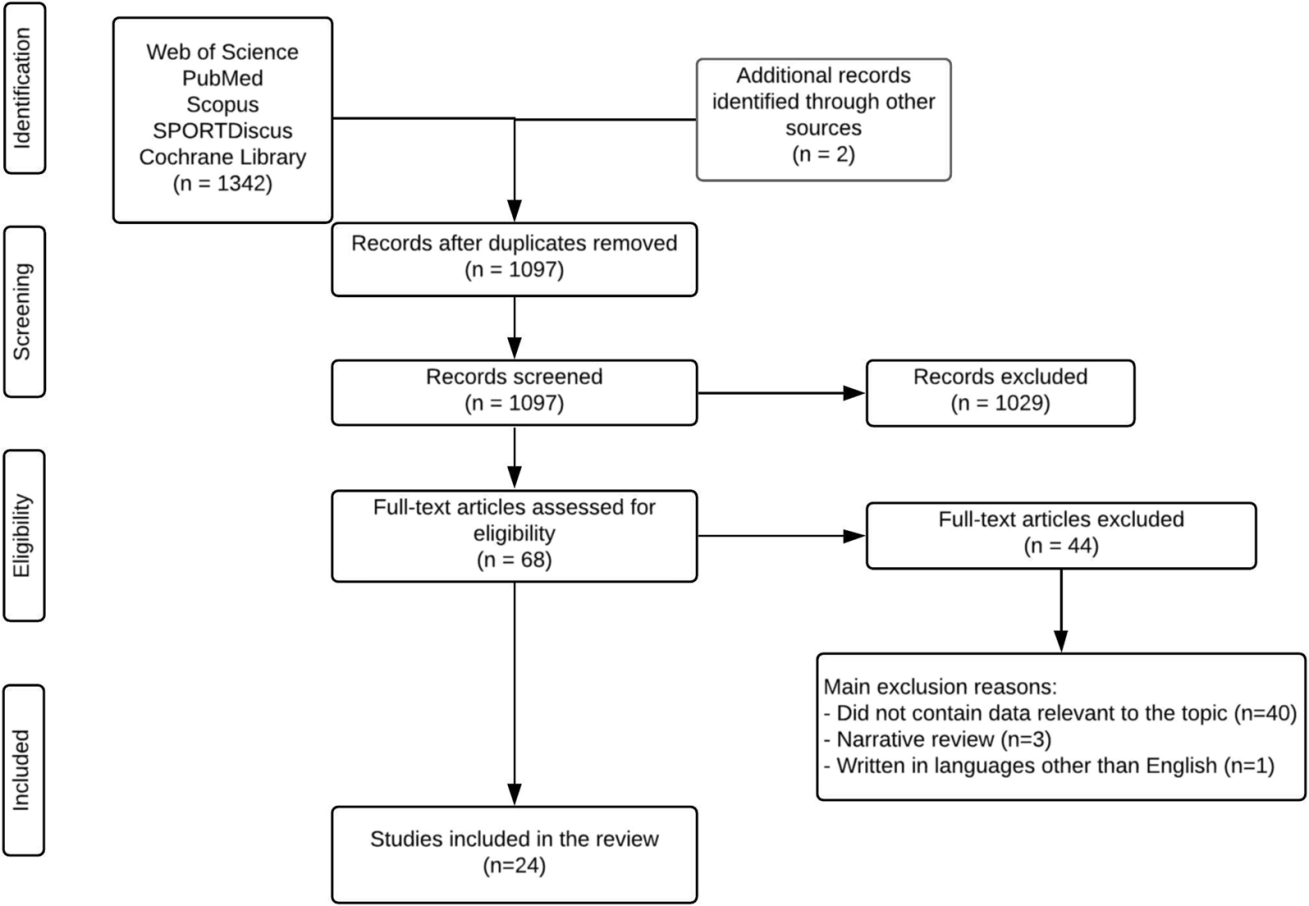
Flow chart of the procedures used for the article search

A total of 1342 references were identified in the databases, and 2 additional references were identified through other sources. These data were then exported to reference manager software (EndNoteTM X9, Clarivate Analytics, Philadelphia, PA, USA). Any duplicates (247 references) were eliminated either automatically or manually. The remaining 1097 articles were then screened for relevance based on their title and abstract, resulting in 1029 studies being eliminated from the database. The full text of the remaining 68 articles was examined in more detail; 44 were rejected because they did not meet the inclusion criteria. At the end of the screening procedure, 24 articles were selected for in-depth reading and analysis (Fig. 1). The main factor for study exclusion (*n* = 40) was the lack of relevance to the research topic of this umbrella review. Other studies were excluded because they were narrative reviews (*n* = 3) or were written in languages other than English (*n* = 1). Of the 24 papers included in this umbrella review, 21 were systematic reviews, and 3 were meta-analyses. The chronological analysis of the articles considered in this review evidenced the recent developments in this area of research, highlighting that 77% of the studies were published within the last 2 years (i.e. from years 2019 to 2020), and the oldest systematic reviews were published 6 years ago.

### Quality of the Studies

The overall methodological quality of the 24 included papers is summarized in Table 1. Based on the rating of overall confidence obtained using AMSTAR-2 [15], the overall confidence in the results of 12 (50%) reviews was rated as “Critically low”. Additionally, the confidence in eight reviews was “Low” (33.3%), and the confidence in three articles was “Moderate” (12.5%). Only one of the 24 reviews (4.2%) was rated as “High”. Our analysis revealed very low confidence in the results of most systematic reviews of match analysis in team ball sports, based on the AMSTAR-2 quality rating criteria. These results are similar to those found in other research areas [17]. For this reason and because match analysis is a recent sports sciences research topic, we did not exclude any of the studies from further analysis based on the quality assessment. One of the advantages of this study is that it proposes research solutions that allow investigators to increase the quality of systematic reviews and meta-analyses on match analysis.

**Table 1:** AMSTAR 2 assessment of each included systematic review

Study	AMSTAR 2—ITEMS																Overall items
	1	2	3	4	5	6	7	8	9	10	11	12	13	14	15	16	
Courel-Ibáñez et al. [18]	Yes	No	No	Yes	Yes	No	No	PY	No	No	No MA	No MA	No	No	No MA	Yes	Critically low
Maimón et al. [19]	Yes	No	No	Yes	No	No	No	Yes	No	No	No MA	No MA	No	No	No MA	Yes	Critically low
Reina et al. [20]	Yes	No	No	Yes	No	No	Yes	PY	PY	No	No MA	No MA	No	No	No MA	Yes	Critically low
Medeiros et al. [21]	Yes	No	Yes	Yes	No	No	No	PY	No	No	No MA	No MA	No	No	No MA	Yes	Critically low
Bujalance-Moreno et al. [22]	Yes	No	Yes	Yes	Yes	No	No	PY	PY	No	No MA	No MA	Yes	No	No MA	Yes	Critically low
Clemente and Sarmento [23]	Yes	No	No	Yes	Yes	Yes	Yes	PY	PY	No	No MA	No MA	Yes	No	No MA	Yes	Low
Clemente et al. [24]	Yes	No	No	Yes	Yes	Yes	Yes	PY	PY	No	No MA	No MA	Yes	No	No MA	Yes	Low
Field et al. [25]	Yes	No	No	Yes	Yes	Yes	Yes	PY	PY	Yes	No MA	No MA	Yes	No	No MA	Yes	Low
Goes et al.[26]	Yes	No	No	Yes	No	No	Yes	PY	No	No	No MA	No MA	No	No	No MA	Yes	Critically low
Low et al. [3]	Yes	No	No	No	Yes	Yes	Yes	PY	PY	No	No MA	No MA	Yes	Yes	No MA	Yes	Critically low
Sarmento et al. [27]	Yes	No	No	No	No	No	No	PY	No	No	No MA	No MA	Yes	No	No MA	Yes	Critically low
Sarmento et al. [28]	Yes	No	No	Yes	Yes	Yes	Yes	PY	PY	No	No MA	No MA	Yes	Yes	No MA	Yes	Low
Sarmento et al. [5]	Yes	No	No	Yes	Yes	Yes	Yes	PY	PY	No	No MA	No MA	Yes	Yes	No MA	Yes	Low
Vieira et al. [29]	Yes	Yes	No	Yes	Yes	Yes	Yes	PY	PY	No	No MA	No MA	Yes	No	No MA	Yes	Moderate
Agras et al. [30]	Yes	No	No	Yes	No	No	No	PY	No	No	No MA	No MA	No	No	No MA	No	Critically low
Rico-González et al. [31]	Yes	No	No	Yes	Yes	No	Yes	PY	PY	No	No MA	No MA	Yes	No	No MA	Yes	Low
Ferrari et al. [32]	Yes	No	No	Yes	Yes	Yes	Yes	PY	PY	No	No MA	No MA	Yes	No	No MA	Yes	Low
Ferraz et al. [33]	Yes	No	No	Yes	Yes	Yes	Yes	PY	PY	No	No MA	No MA	Yes	No	No MA	Yes	Low
Colomer et al. [34]	Yes	No	No	No	No	No	Yes	PY	No	No	No MA	No MA	No	No	No MA	Yes	Critically low
Glassbrook et al. [35]	Yes	Yes	No	Yes	Yes	No	Yes	Yes	PY	No	Yes	Yes	Yes	Yes	Yes	Yes	Moderate
Hausler et al.[36]	Yes	No	No	Yes	Yes	Yes	Yes	Yes	Yes	No	Yes	Yes	Yes	Yes	Yes	Yes	Moderate
Silva et al. [37]	Yes	No	No	Yes	Yes	No	Yes	PY	No	No	No MA	No MA	Yes	No	No MA	No	Critically low
Harper et al. [38]	Yes	Yes	Yes	Yes	Yes	Yes	Yes	Yes	Yes	No	Yes	Yes	Yes	Yes	Yes	Yes	High
Fernández-Espínola et al. [39]	Yes	No	No	Yes	Yes	No	Yes	PY	PY	No	No MA	No MA	No	No	No MA	Yes	Critically low

PY—Partial Yes; No MA—No Meta-analysis; Description of AMSTAR-2 Items: 1—Did the research questions and inclusion criteria for the review include the components of PICO?; 2—Did the report of the review contain an explicit statement that the review methods were established prior to the conduct of the review and did the report justify any significant deviations from the protocol?; 3—Did the review authors explain their selection of the study designs for inclusion in the review?; 4—Did the review authors use a comprehensive literature search strategy?; 5—Did the review authors perform study selection in duplicate?; 6—Did the review authors perform data extraction in duplicate?; 7—Did the review authors provide a list of excluded studies and justify the exclusions?; 8—Did the review authors describe the included studies in adequate detail?; 9—Did the review authors use a satisfactory technique for assessing the risk of bias (RoB) in individual studies that were included in the review?; 10—Did the review authors report on the sources of funding for the studies included in the review?; 11—If meta-analysis was performed did the review authors use appropriate methods for statistical combination of results?; 12—If meta-analysis was performed, did the review authors assess the potential impact of RoB in individual studies on the results of the meta-analysis or other evidence synthesis?; 13—Did the review authors account for RoB in individual studies when interpreting/discussing the results of the review?; and 14—Did the review authors provide a satisfactory explanation for, and discussion of, any heterogeneity observed in the results of the review?; 15—If they performed quantitative synthesis did the review authors carry out an adequate investigation of publication bias (small study bias) and discuss its likely impact on the results of the review?; 16—Did the review authors report any potential sources of conflict of interest, including any funding they received for conducting the review?

Of note, all included studies were conducted after the PRISMA statement was published in 2009 [13]. One possible reason for this low level of confidence is the lack of protocol registration in most of the analysed articles (88%). Even though PROSPERO does not accept records of protocols that do not include health-related outcomes, researchers in this scientific area have other platforms (e.g. Campbell, Cochrane, Inplasy, Open Science Framework) on which they can register their protocols before starting their reviews.

Additionally, only three reviews explained their selection of the study designs for inclusion in the review. The authors also commonly failed to perform study selection (33%) and data extraction in duplicate (54%). Approximately 33% of the included papers did not use a satisfactory technique for assessing the risk of bias (RoB) of individual studies included in the review. Several tools have been developed over the past few years to assess this aspect of such articles beyond “traditional” tools dedicated to studies related exclusively to health outcomes analyses. In this sense, authors studying match analysis can use these available tools to analyse the RoB of studies and discuss the results of their reviews. Our study highlights the need to improve the quality of systematic reviews in the field of performance analysis in sports.

### Qualitative Synthesis

The selected systematic reviews mentioned no criteria limiting the type of experimental design adopted by the investigations. The number of studies included in these reviews ranged from 11 [25] to 79 [30] (Table 2). There is a clear predominance of cross-sectional observational studies; thus, longitudinal studies are warranted. Furthermore, randomized trials and randomized controlled trials are lacking in cross-sectional studies. Finally, only 14 of the 24 systematic reviews assessed the research quality and/or RoB [3, 5, 20, 22–25, 28, 29, 33, 35, 36, 38, 39], which concurs with the previous evaluation of the quality of studies through AMSTAR.

**Table 2:** Synthesis of the reviewed studies

Study	Purpose of the study and context of analysis	Outcomes
Courel-Ibáñez et al. [18]	*Sport(s):* Basketball *Main goal*: Assess common research topics, main findings and shortcomings in terms of analysis of collective behaviour *Type of publications included*: peer-reviewed journal articles with no limitations in experimental design *No. studies included*: 45 *Context of analysis*: formal matches *Analyses*: descriptive, comparative and “predictive” *Competitive level*: Non-professional, professional, youth *Age groups*: Adults and young players (U12-onwards) *Sex*: Mostly men (85.7%)	*Levels of analysis*: predominantly individual (molecular level), but also dyadic (microlevel), group (mesolevel) and team dynamics (macrolevel) *Performance dimensions*: tactical, technical/biomechanical, physical/physiological *Within tactical dimension*: game patterns, game pace, effectiveness, spatial differentiation, temporal features of actions, game phases *Functional groups:* playing roles *Main findings:* Lack of studies exploring dynamic takes on tactical behaviour and an absence of longitudinal designs. Information on how contextual factors impact on match performance *Match context:* match location (one study suggested that defensive strategies may be influenced by this factor), match period (one study suggested that losing teams may apply more defensive pressure in the two last periods of the match)
Maimón et al. [19]	*Sport(s)*: Basketball *Main goal*: Analyse the basketball pass and the factors influencing its learning and performance *Type of publications* *included*: peer-reviewed journal articles with no limitations in experimental design *No. studies included*: 37 *Context of analysis*: formal matches, small-sided games and training drills *Analyses*: descriptive, comparative and “predictive” *Competitive level*: from beginners to elite level *Age groups*: from U11 to adults *Sex*: men (21), both (13), women (3)	*Levels of analysis:* individual, team *Performance dimensions*: tactical, decision-making, physical/physiological, technical/biomechanical, psychosocial *Within tactical dimension*: game patterns, uncertainty *Functional groups:* playing roles *Main findings:* Skill assessments should be performed under uncertain and variable conditions. Novel and random tasks may potentiate transfer from training to competition. Practicing under pressure may reduce choking in competition. Physical conditioning is paramount for maintaining skill proficiency during matches. SSGs are useful for improving passing skills *Match context*: unreported
Reina et al. [20]	*Sport(s):* Basketball *Main goal:* Analyse the state of the art on internal and external loads in women’s basketball *Type of publications included:* peer-reviewed journal articles with no limitations in experimental design *No. of studies included:* 26 *Context of analysis:* formal matches and training sessions *Analyses*: descriptive, comparative and “predictive” *Competitive level*: from state to international level *Age groups*: from U14 to adults *Sex*: women	*Levels of analysis:* Individual *Performance dimensions*: Physical/physiological, technical/biomechanical *Main findings:* most studies focused on adult players (66.7%), with comparatively little research on age groups, especially below U18. Most research focus on training or competition, with only a select few analysing both training and competition (< 15%). Studies with international-level women basketball players represented < 5% of the sample. > 55% of studies analysed metrics of both internal and external load *Match context:* unreported
Medeiros et al. [21]	*Sport(s):* Beach volleyball *Main goal:* review the literature on match analysis in beach volleyball *Type of publications included:* peer-reviewed journal articles with observational methodology *No. of studies included:* 18 *Context of analysis:* formal match *Analyses*: descriptive, comparative and “predictive” *Competitive level*: national elite, international elite *Age groups*: adults *Sex*: men and women	*Levels of analysis:* Individual, team *Performance dimensions*: Tactical physical/physiological *Within tactical dimension*: game patterns, effectiveness *Main findings:* research has slowly evolved towards more complex and “predictive” models. Most game levels have not been studied yet. Contextual variables such as match status and quality of opposition have not been considered. Rule changes induced changes in game dynamics and physiological demands *Match context*: unreported
Bujalance-Moreno et al. [22]	*Sport(s):* Football (soccer) *Main goal*: describe the acute and chronic adaptations *Type of studies included*: peer-reviewed journal articles with no limitations in experimental design *No. studies included*: 53 *Context of analysis*: small-sided games *Analyses:* descriptive and comparative *Competitive level*: amateur and professionals *Age*: above 16 years-old *Sex*: men	*Levels of analysis*: only individual (molecular level) *Performance dimensions*: technical/biomechanical and physical/physiological *Main findings:* heart rate, blood lactate and rate of perceived exertion were the primary physiological outcomes; distances covered at different speed thresholds were the main primary outcomes in physical demands. Considering the technical/biomechanical dimensions it was found that passes and shots occurred more often in smaller pitch dimensions; both intermittent and continuous regimens resulted in similar improvements in technical aspects *Match context*: unreported
Clemente and Sarmento [23]	*Sport(s):* Football *Main goal*: analyse the effects of task constraints on technical actions *Type of studies included*: peer-reviewed journal articles with no limitations in experimental design *No. studies included*: 37, *Context of analysis*: small-sided games *Analyses*: descriptive and comparative *Competitive level*: all *Age*: all *Sex*: men	*Levels of analysis*: only individual (molecular level) *Performance dimensions*: technical/biomechanical *Main findings:* pass, receives, turn, dribbles, header, tackle, block, interception, shots (main findings revealed that smaller formats and smaller pitches meaningfully increased the individual actions. Moreover, free play and the absence of goalkeepers also contributed to meaningfully increase the number of technical actions. Finally, age also influenced the accuracy of actions). The effects of coaches’ intervention also revealed significant influence on attacking and defensive actions *Match context*: unreported
Clemente et al. [24]	*Sport(s)*: Football *Main goal*: analyse the effects of task constraints on tactical behaviour and collective dynamics *Type of studies included*: peer-reviewed journal articles with no limitations in experimental design *No. studies included*: 34 *Context of analysis*: small-sided games *Analyses*: descriptive and comparative *Competitive level*: all *Age*: all *Sex*: men	*Levels of analysis*: predominantly individual (molecular level), but also dyadic and group (mesolevel) *Performance dimensions*: tactical *Within tactical dimension*: group dynamics *Main findings:* team’s centre, distance between players and team’s centre, area variables (e.g. team width and length, surface area, stretch index), space between players (e.g. ellipse areas, effective area of play). Main evidence revealed that older players increase the exploration of the width and length. Numerical imbalances contribute to more stable defensive dynamics. Larger pitches increased variability of movements *Match context*: unreported
Field et al. [25]	*Sport(s):* Football *Main goal*: summarize the physical demands during extra-time, as well as technical actions *Type of studies included*: peer-reviewed journal articles with no limitations in experimental design *No. studies included*: 11 *Context of analysis*: formal matches *Analyses*: descriptive and comparative *Competitive level*: all *Age*: adults *Sex*: men	*Levels of analysis*: only individual (molecular level) *Performance dimensions*: technical/biomechanical, physical/physiological *Main findings:* physical demands (e.g. distances covered at different speed thresholds, number of accelerations and decelerations) and physiological (e.g. blood glucose, lactate, creatine kinase, potassium, haemoglobin, insulin, glycerol, heart rate). Main findings revealed decremental effects of extra-time on physical performance (e.g. distance covered per minute) and that recovery is longer in matches with extra-time. Shooting performance, passes, dribbles and ball in play. Results revealed that technical performance reduced in extra-time comparing to regular period. Additionally, a positive effect of carbohydrate intake on technical performance was observed during extra-time *Match context:* match period (in the extra 30 min, less distance is covered by the players than during the regular 90 min)
Goes et al. [26]	*Sport(s):* Football *Main goal*: describe the evidence about tactical behaviour measured by position data analysis *Type of studies included*: peer-reviewed journal articles with no limitations in experimental design *No. studies included*: 73 *Context of analysis*: formal matches *Analyses*: descriptive, comparative and “predictive” *Competitive level*: all *Age*: all *Sex*: not defined	*Levels of analysis*: predominantly group (mesolevel) and, but also, team dynamics (macrolevel) *Performance dimensions*: tactical *Within tactical dimension*: team and group dynamics *Within tactical dimension*: set pieces (did not influence the final result) *Main findings:* space control, spread/surface, length/width and centroids. Results indicated that the majority of the studies used centroids and spread/surface measures. However, pending the area of intervention, the conclusions are different, and this should be considered in further studies *Match context:* match period (Here, a different approach was used, analysing time windows linked to given match events, i.e. what happened after or before certain key actions)
Low et al. [3]	*Sport(s):* Football *Main goal:* summarize the research on collective tactical behaviours in football *Type of publications included:* peer-reviewed journal articles with no limitations in experimental design *No. of studies included:* 77 *Context of analysis:* formal matches and small-sided games *Analyses*: descriptive, comparative and “predictive” *Competitive level*: all *Age*: all *Sex*: men and women	*Levels of analysis*: individual, group (mesolevel) and, team dynamics (macrolevel) *Performance dimensions*: tactical *Within tactical dimension*: team and group dynamics *Functional groups:* playing roles *Main findings:* Main outcomes were classified in position, distance, playing spaces and numerical relations. These outcomes were analysed by linear and nonlinear statistical methods. In the case of nonlinear statistics, it was used to identify regularities, synchronization tendencies and also to predict events. Specific comparators such as tactical expertise, age, numerical relationships or playing roles affected the outcomes *Match context:* unreported
Sarmento et al. [27]	*Sport(s):* Football *Main goal*: describe the evidence about match analysis *Type of studies included*: peer-reviewed journal articles with no limitations in experimental design *No. studies included*: 53 *Context of analysis*: formal matches *Analyses*: descriptive, comparative and “predictive” *Competitive level*: all *Age*: all *Sex*: men	*Levels of analysis*: individual (molecular level) *Performance dimensions*: technical/biomechanical, physical/physiological *Functional groups:* playing roles *Within tactical dimension*: set pieces (did not influence the final result) *Main findings:* physical demands (distances covered at different speed thresholds) are influenced by competitive level, success of the team and contextual factors. Differences in physical demands were found between playing positions. Shots, passes, involvements with the ball (can be influenced by the type of attack as well as by the context of recovery). Moreover, style of ball possession differentiates winning from losing teams *Match context:* match location (there seems to be a home advantage, with the home team having increased likelihood of winning, greater efficacy in most performance indicators, and also commit fewer fouls), match period (performance in 2nd half tends to decrease, but mostly if the 1st half demanded high total distances covered) and quality of opposition (difficult to compare, since different research teams used distinct strategies to analyse this factor. However, there seems to be different tactical behaviours when against strong opponents vs. weaker opponents)
Sarmento et al. [28]	*Sport(s):* Football *Main goal*: describe the effects of task constraints on physiological, physical, technical and tactical outcomes *Type of studies included*: peer-reviewed journal articles with no limitations in experimental design *No. studies included*: 77 *Context of analysis*: small-sided games *Analyses*: descriptive and comparative *Competitive level*: all *Age*: all *Sex*: men	*Levels of analysis*: predominantly individual (molecular level), but also group (mesolevel) *Performance dimensions*: tactical, technical/biomechanical, physical/physiological *Within tactical dimension*: team group dynamics *Main findings:* physiological (heart rate, blood lactate concentrations and rate of perceived exertion) and physical outcomes (distances covered at different speed thresholds). Smaller pitches and formats were conducive to higher physiological stimulus while bigger pitches increased physical demands. Considering the decision-making, tactical behaviour changed based on the type of games; namely more individual attacking behaviours occurred in smaller formats. On the other hand, bigger formats were conducive to increases in tactical principles related to unity. In the case of technical/biomechanical analysis passes, tackles, headers, dribbles, crosses and shots were observed. Main evidence revealed that smaller formats and pitches were conducive to increases in the number of individual actions *Match context:* match status (suggestions that balanced and unbalanced scenarios promote different tactical behaviours, but the collected data are sparse and heterogeneous), match location (Home advantage is reported for several performance indicators), match period (general decrements in performance from the 1st to the 2nd half. Even greater decrements occur when extra time has to be played)
Sarmento et al. [5]	*Sports(s):* Football *Main goal*: systematize the main evidence about match analysis from 2012 to 2016 *Type of studies included*: peer-reviewed journal articles with no limitations in experimental design *No. studies included*: 77 *Context of analysis*: formal matches *Analyses:* descriptive and comparative *Competitive level*: all *Age*: all *Sex*: men *Other:* impact of altitude and heat stress on physical performance	*Levels of analysis*: individual (molecular level), but also dyadic, group (mesolevel) and team dynamics (macrolevel) *Performance dimensions*: tactical, technical/biomechanical, physical/physiological *Functional groups:* playing roles *Within tactical dimension*: game phase, game patterns and team/group dynamics, set pieces *Main findings:* team centre, team dispersion and team interaction. Regarding team’s centre, it was possible to identify an in-phase relationship between teams. Dispersion was higher in attacking moments than in defensive ones. Considering the overall interactions, high passing rates were related to an increase in team performance. Considering the physical/physiological dimensions it was found that distances covered at different speed thresholds were not influenced by congested periods. Meaningful differences in physical demands between positions were revealed. Additionally, network centrality measures revealed prominence of midfielder players. Corner kicks, penalty kicks and free kicks. In the case of corner kicks, zonal marking seems to reduce the goals conceded compared to one-to-one marking. Considering the penalty kick, the main factors for accuracy were related with the area for shooting, speed of the ball, situational factors and using strategies against the goalkeeper. Considering the free kicks, it was found a very small percentage of goals scored, however such rate can be affected by different factors (e.g. location, interruption time, distance to defensive wall, number of players in the wall) *Match context*: match status, match location, match period (extra-time)
Vieira et al. [29]	*Sport(s):* Football *Main goal*: summarize the evidence about match running performance *Type of studies included*: peer-reviewed journal articles with no limitations in experimental design *No. studies included*: 50 *Context of analysis*: formal matches *Analyses*: descriptive, comparative and “predictive” *Competitive level*: amateur *Age*: young *Sex*: men *Other:* altitude and pitch surface	*Levels of analysis*: individual (molecular level) *Performance dimensions*: physical/physiological *Functional groups:* playing roles *Main findings:* Physical demands were analysed (e.g. peak velocity; different speed thresholds; meters per minute). Relationships between biochemical measures, recovery and running performance were observed. Congested fixture seems to impair match running performance. Residual differences were found between outfield players; however interaction with age category was inconsistent *Match context:* match period (Contrasting results regarding comparisons between halves, although most studies show no changes or decrements from the 1st to the 2nd half)
Agras et al.[30]	*Sport(s)*: Futsal *Main goal*: describe the main findings in time-motion and notational analysis *Type of studies included*: peer-reviewed journal articles with no limitations in experimental design *No. studies included*: 79 *Context of analysis*: formal matches *Analyses:* descriptive and comparative *Competitive level*: all *Age:* all *Sex:* mostly men, but also women	*Levels of analysis*: predominantly individual (molecular level), but also group (mesolevel) and team dynamics (macrolevel) *Performance dimensions*: tactical, technical/biomechanical, physical/physiological *Within tactical dimension*: game phase *Within tactical dimension*: set pieces (did not influence the final result) *Main findings:* Greater technical efficacy was not related with better team’s results. Regarding physical demands, decreases were found in second half (e.g. distance covered). Best teams presented higher efficiency in the attack; counterattack raised predominantly from defensive field using group actions *Match context*: match period. One study mentioned that during the 2nd period the intensity of actions falls. Another study mentioned that offensive numerical superiority increased in the fourth period of play. A few studies suggested that more goals were scored in the latter periods of the match
Rico-González et al. [31]	*Sport(s):* Futsal *Main goal*: describe the main position-based measures to assess team behaviour *Type of studies included*: peer-reviewed journal articles with no limitations in experimental design *No. studies included*: 12 *Context of analysis*: formal matches and small-sided games *Analyses:* descriptive and comparative *Competitive level*: national elite, international elite *Age groups*: youth and adults *Sex*: men	*Levels of analysis*: individual, group (mesolevel) and team dynamics (macrolevel) *Performance dimensions*: tactical *Within tactical dimension*: team and group dynamics *Main findings:* most of the analysed situations were sub-group dynamics within the match. The main outcomes assessed were related to geometrical centre relationships between teams and also distance relationships between players and the ball. Moreover, the occupied area was also inspected using dominant areas. The variability of behaviours was measured using relative-phase and entropy to identify complexity and regularity *Match context*: unreported
Ferrari et al. [32]	*Sport(s)*: Handball *Main goal*: describe the main research tendencies in match analysis *Type of studies included*: peer-reviewed journal articles with no limitations in experimental design *No. studies included*: 28 *Context of analysis*: formal matches *Analyses*: descriptive, comparative and “predictive” *Competitive level*: amateur or professional *Age*: adult *Sex*: men	*Levels of analysis*: predominantly individual (molecular level), but also team dynamics (macrolevel) *Performance dimensions*: tactical, technical/biomechanical *Within tactical dimension*: game phase *Functional groups:* playing roles *Within tactical dimension*: the 6- and 9-m throws had great relevance in offensive teams *Main findings:* attack (winning teams make continuous and short attacks; losing teams perform long positional attacks and attacks based on individual attempts) and defensive (the strategy of stopping offensive actions by physical contact, or avoiding fouls or just focusing in the interception of the ball were not particular favourable in the defensive success). Considering technical/biomechanical dimensions the number of goals scored was the main reason for the team’s success and weak defensive actions were related with more goals conceded *Match context*: match status (one study showed that home advantage seems more prominent for balanced matches), match location (a few studies suggest a home advantage effect in terms of effectiveness and likelihood of winning the matches) and match period (one study suggested that more goals seem to be scored in the latter periods of the match. Another stated that time-outs are more effective if requested in the first 20 min of each game period)
Ferraz et al. [33]	*Sport(s):* Rink hockey *Main goal*: summarize the main evidence about player’s profile and game demands *Type of studies included*: peer-reviewed journal articles with no limitations in experimental design *No. studies included*: 19 *Context of analysis*: formal matches *Analyses*: descriptive and comparative *Competitive level*: all *Age*: all *Sex*: men	*Levels of analysis*: predominantly individual (molecular level), but also team dynamics (macrolevel) *Performance dimensions*: tactical, technical/biomechanical, physical/physiological *Within tactical dimension*: game phase; game patterns *Functional groups:* playing roles *Main findings:* greater frequency of units in indirect attack than in counterattack or quick transition. Attack (greater number of shooting comes from central zones; almost 50% of offensive actions lead to an attempt to score; only 3% of the attempts results in a goal; match status is particularly relevant in the goalkeeper’s performance). Technical and tactical actions are involved in longer patterns of play *Match context:* The authors refer to match status, but actually should have said match period: one paper suggested that goalkeepers are less effective in the 2nd half of the match, probably due to fatigue
Colomer et al. [34]	*Sport(s):* 15-a-side rugby union *Main goal:* Analyse the state of the art of performance analysis research in professional rugby union *Type of publications included:* peer-reviewed journal articles with observational methodology *No. of studies included:* 41 *Context of analysis:* formal match *Analyses*: descriptive, comparative and “predictive” *Competitive level*: professional *Age groups*: adults *Sex*: men	*Levels of analysis:* individual, team *Performance dimensions*: tactical *Within tactical dimension*: game patterns, special plays (set pieces) *Main findings:* Most studies did not provide context relating to match location, quality and type of opposition and other potentially relevant information. Only 5 of 41 studies accounted for multiple contextual variables. Only 7 articles provided operational definitions for analysing the variables *Match context*: match location, match period and, in < 20% of the studies, quality of opposition. The majority of studies did not provide such information, though. The review does not detail how this was handled by each specific study
Glassbrook et al. [35]	*Sport(s):* Rugby league *Main goal:* Analyse studies investigating the physical demands of professional rugby league matches *Type of publications included:* peer-reviewed journal articles with observational methodology *No. of studies included:* 30 *Context of analysis:* Formal match *Analyses*: descriptive, comparative and “predictive” *Competitive level*: Professional *Age groups*: Adults *Sex*: Men	*Levels of analysis:* Individual *Performance dimensions*: Physical/physiological *Functional groups:* Playing roles *Main findings:* Physical demands of Rugby Union vary depending on the playing role, but not for all variables *Match context:* Unreported
Hausler et al. [36]	*Sport(s):* Rugby league *Main goal:* review use of GPS and microtensor technology to quantify player activity profiles in match-play *Type of publications included:* peer-reviewed journal articles with observational methodology *No. of studies included:* 27 *Context of analysis:* Formal match *Analyses*: descriptive, comparative and “predictive” *Competitive level*: from junior to elite *Age groups*: junior and adults *Sex*: Men	*Levels of analysis:* Individual *Performance dimensions*: Physical/physiological *Functional groups:* Playing roles *Main findings:* most studies focus on the adult elite level, with sub-elite, amateur and junior levels being under-represented. Activity profiles vary depending on playing roles and level of practice. Comparisons of high-speed movements are difficult because of inconsistent description across studies *Match context:* match period (five studies reported on pacing strategies changing depending on time-period, with the greatest decrements in performance in the last two periods of the match)
Silva et al. [37]	*Sport(s):* Volleyball *Main goal:* Analyse the literature on match analysis in volleyball *Type of publications included:* peer-reviewed journal articles with observational methodology *No. of studies included:* 34 *Context of analysis:* formal match *Analyses*: descriptive, comparative and “predictive” *Competitive level*: amateur and professional (national elite and international) *Age groups*: adults *Sex*: mostly men, but also women	*Levels of analysis:* Individual, team *Performance dimensions*: tactical, technical/biomechanical *Within tactical dimension*: game patterns, effectiveness, decision-making *Functional groups:* Playing roles *Main findings:* The quality of each action is deeply related with the effectiveness of previous actions. Considerable focus on effectiveness of actions, but not necessarily on the qualitative analysis of the skills. Lack of understanding of game dynamics and patterns, since the focus is on actions’ effectiveness *Match context:* match period (one study showed strategic differences in the use of the serve and attack depending on match period), match status (one study showed strategic differences depending on match status) and quality of opposition (one study showed strategic differences depending on quality of opposition)
Harper et al. [38]	*Sport(s):* American football, Australian football, hockey, rugby league, rugby sevens, rugby union, football *Main goal:* Compare high and very high intensity acceleration vs deceleration demands during match-play in elite team sports *Type of publications included:* peer-reviewed journal articles with observational methodology *No. of studies included:* 19 *Context of analysis: * formal match *Analyses*: descriptive, comparative and “predictive” *Competitive level*: elite *Age groups*: adults *Sex*: Men	*Levels of analysis:* Individual *Performance dimensions*: Physical/physiological *Main findings:* In American football, high and very high intensity accelerations are more frequent than decelerations. Conversely, in all other sports there is a greater frequency of high and very high intensity decelerations compared to accelerations *Match context:* match period (when comparing the 2nd game period to the first, there is a small decrease in high and very high intensity accelerations and decelerations)
Fernández-Espínola et al. [39]	*Sport(s)*: Mostly football, but also basketball, hockey, handball, rugby *Main goal:* Assess the role of SSGs as a tool for teaching team sports *Type of publications included:* peer-reviewed journal articles without limitations in terms of experimental design *No. of studies included:* 47 *Context of analysis:* small-sided games *Analyses*: descriptive, comparative and “predictive” *Competitive level*: sports initiation *Age groups*: U18 *Sex*: Mostly men. Unreported in several studies. Only one study specifically with girls *Other:* a few selected studies analysed the effect of maturation, duration, coach’s presence, among other factors	*Levels of analysis:* individual, group, team *Performance dimensions*: tactical, technical/biomechanical, decision-making *Within tactical dimension*: game patterns, relationships between tactical actions and skill, set pieces, manipulation of task constraints *Main findings:* SSGs are useful for teaching team sports at young ages. Besides increased number of technical actions, constraints should be manipulated to fully explore the potentialities of SSGs. Among the most relevant manipulations are: number of players, size of pitch area, playing rules, duration and presence of the coach *Match context*: unreported

SR, systematic review; SSG, small-sided game|review

#### Demographic Analysis of Studies

There is a clear overrepresentation of studies with male participants. Of the 24 included systematic reviews, 15 (62.5%) exclusively analysed research on male athletes. Only one review (4.5%) focused exclusively on female athletes [20]. Another review did not report the participants’ sex [26]. Of the remaining seven reviews (29.2%), only one presented a balanced inclusion of studies on men and women [21], while the other six studies were disproportionately represented by male participants [3, 18, 19, 30, 37, 39]. Considering the main participant populations in the studies, seven reviews (29.2%) [21, 25, 32, 34, 35, 37, 38] exclusively focused on adults and 15 (62.5%) had no age restrictions and, thus, included both youth and adult athletes [3, 5, 18–20, 22–24, 26–28, 30, 31, 33, 36]. Reviews by Fernández-Espínola et al. [39] and Vieira et al. [29] focused exclusively on young players.

Furthermore, football (soccer) is overrepresented among the team sports sampled, as 10 systematic reviews (41.7%) exclusively focused on this sport [3, 5, 22–29]. Three systematic reviews focused on rugby codes, one focused on rugby union [34], and two focused on rugby league [35, 36]. Three systematic reviews investigated basketball [18–20], and another two were conducted on futsal [30, 31]. Handball [32], rink hockey [33], beach volleyball [21], and indoor volleyball [37] were each the focus of one review. Two systematic reviews analysed multiple sports. Fernández-Espínola et al.’s review [39] mostly included articles on football but also included some on basketball, hockey, handball, and rugby. Harper et al.’s [38] review included studies on American football, Australian Rules football, hockey, rugby league, rugby union, rugby sevens, and football. In terms of game format, five (20.8%) exclusively analysed performance in small-sided games [22–24, 28, 39], two analysed both small-sided and full-sided games [3, 31], and the remaining 17 (70.8%) exclusively analysed full-sided matches.

**Fig. 2:**
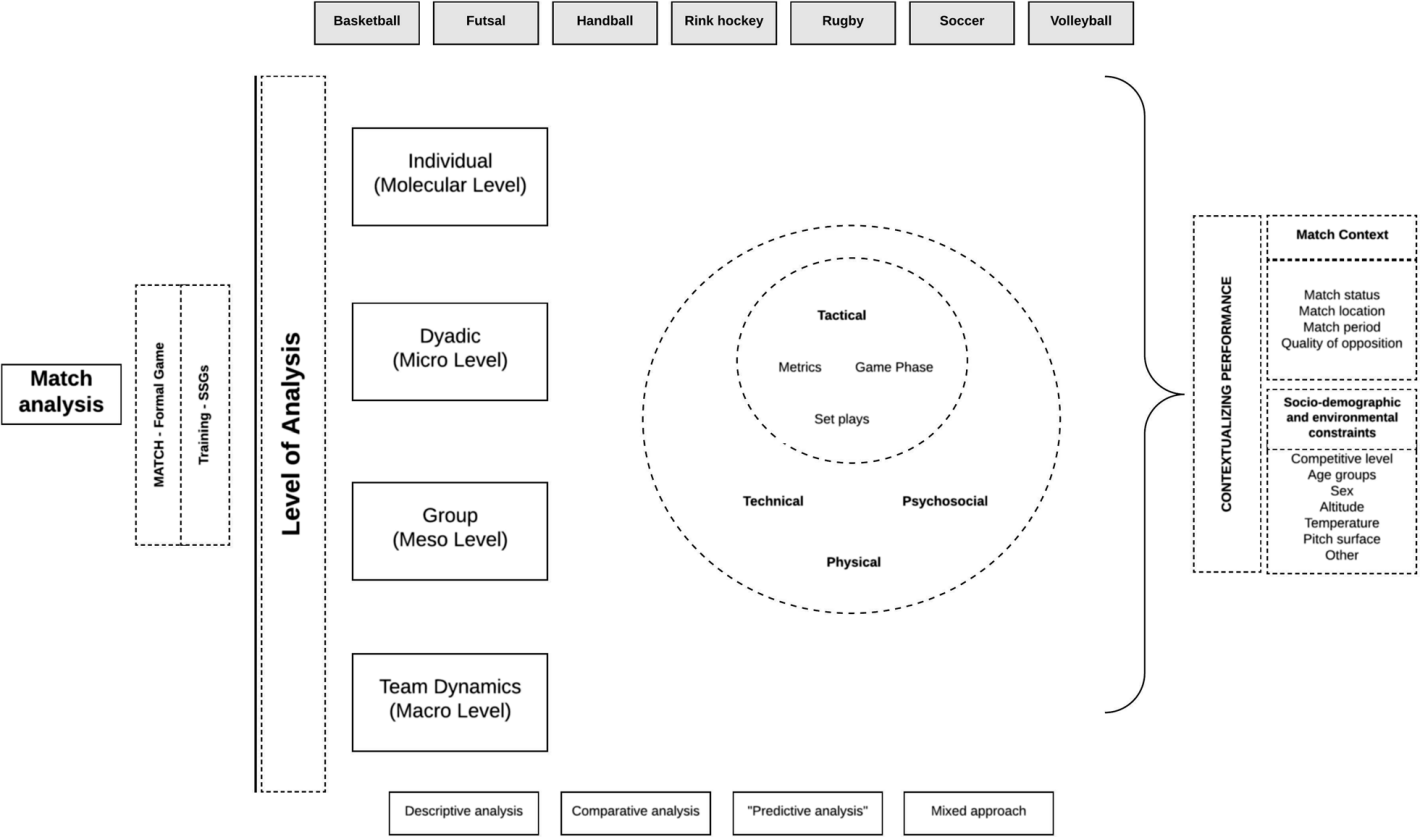
Scopes of talent identification and development

The ecological dynamics theoretical framework argues that the dynamic performer–environment relationships are the most relevant scale for understanding behaviour [40]. Skill acquisition and expert performance in sports should consider both the macro- and microstructure of practice [41]. Interestingly, despite focusing on team sports, 23 of the 24 systematic reviews (95.8%) reported findings at the molecular (i.e. individual) level of analysis, with the exception of the review by Goes et al. [26]. In comparison, only 13 (54.2%) reviews provided analyses at the macrolevel (i.e. team) [3, 5, 18, 19, 21, 26, 30–34, 37, 39]. The mesolevel of analysis (i.e. subgroup) was reported in only nine reviews [3, 5, 18, 24, 26, 28, 30, 31, 39]. A microlevel (i.e. dyadic) analysis was performed in only three reviews [5, 18, 24]. Only two reviews included all four levels (i.e. from molecular to macro) of analysis [5, 18] (Fig. 2).

With respect to the dimensions of analysis, tactical aspects of performance were analysed in 15 reviews, technical aspects were reviewed in 14 reviews, physical factors were considered in 15 reviews, and only one review presented data on psychosocial variables [19]. Ten of the 24 reviews (41.7%) provided unidimensional analyses rather than multidimensional perspectives on performance. Among the reviews that carried out unidimensional analyses, four reviews analysed only the physiological aspects of performance [29, 35, 36, 38], five reviews focused exclusively on tactical dimensions [3, 24, 26, 31, 34], and one focused solely on the technical (including biomechanical) dimension [23].

Overall, in the selected papers, contextual factors could have been further explored. The mediation of *match period* on performance behaviours was by far the most commonly reported contextual factor, as it was addressed by 11 of the 24 reviews (45.8%) [5, 18, 25–27, 29, 30, 32, 34, 36, 38]. Match location was analysed in five reviews [5, 18, 27, 32, 34]. The effects of *match status* were reported in four reviews, corresponding to 16.7% of the sample [5, 27, 32, 33]. *Quality of opposition* was considered in only two reviews [27, 34]. However, Colomer et al. [34] calculated that < 20% of the papers they reviewed analysed performance according to the quality of opposition. Finally, 11 of the 24 reviews (45.8%) made no reference to performance analysis according to any of these contextual factors [19–24, 28, 35, 39].

## Discussion

This umbrella review of systematic reviews and meta-analyses on performance analysis in team ball sports was conducted to identify current gaps in the literature and make suggestions for future research.

Reviews focused on football represented ~ 41.7% of the sample of studies (*n* = 10), making it the most commonly reviewed sport. Football was also represented in the two multisports reviews [38, 39], meaning it was studied to some extent in 50% of the reviews. Three systematic reviews focused on rugby codes, three focused on basketball, and two focused on futsal. Rink hockey, handball, beach volleyball, and indoor volleyball were represented by one review each. No systematic review has been undertaken for the performance analysis of water polo, ice hockey, or other team sports.

Football is perhaps the most popular sport in the world, and its dominance in performance analysis research appears to reflect that. A quick search of Scopus on 6 August 2020 for articles with “football” in the title provided 11,383 documents. This number is much higher than corresponding values for searches of basketball (4540), volleyball (2110), handball (1299), and water polo (351). On a deeper level, however, sport sciences may be feeding football’s popularity and generating a positive feedback loop at the expense of the other team sports.

### Type of Studies

Only five of the selected reviews explained specific limitations concerning the inclusion criteria related to study designs [21, 34–37]. The main methodological core of the included studies was observational analytics. Some of the original studies included in the systematic reviews presented new measures or specific contexts for testing. However, overall, the studies tended to identify a small number of events or matches in specific tactical analyses, which is reported as a limitation in some of the reviews [3, 24, 31]. Often, studies of tactical analysis are centred on cross-sectional designs [21, 24, 26, 28, 31], although some analyses of cohorts have used longitudinal approaches. On the other hand, studies dedicated to physical performance demands often present outcomes that are repeated between experiments, with these changes focusing heavily on the research question [25, 29, 36, 38].

Future studies should use longitudinal approaches and, if possible, experimental designs; that is, they should employ controlled interventions to identify changes in specific technical, tactical, and physical parameters. This methodological change would increase the range of study designs and use some of the performance outcomes in pre/posttest designs.

All the systematic reviews and meta-analyses included in the present study have considered descriptive and comparative analyses, and several have also addressed predictive analysis [3, 18–21, 26, 27, 29, 32, 34–39]. However, since these studies were cross-sectional, statistical outcomes may not be predictive; they may have merely reflected the idiosyncrasies of the specific samples studied at those specific moments.

Of the reviews included in this umbrella analysis, only three performed meta-analyses [35, 36, 38], all of which were related to physical performance demands. Even though meta-analyses serve simply as supplements to comprehensive systematic reviews, this trend reveals the lack of research questions that may be investigated using meta-analysis techniques. This matter needs to be considered in future studies. In the future, researchers can consider defining clearer research questions seeking to respond to more specific and phenomenon-related objectives. Doing so could provide more evidence and possibilities for synthesis in future reviews and meta-analyses. Additionally, researchers dedicated to this study approach in team sports need to clearly differentiate between systematic reviews, narrative reviews, and integrative reviews.

### Level of Analysis

In our study, four levels of analysis were considered regarding game events: (1) macrolevel (i.e. the team); (2) mesolevel (group); (3) microlevel (dyads), and (4) molecular level (individual). One logical expectation is that most of the analyses would be focused on the team level of analysis (i.e. they dissect the emergence of collective game patterns and synergies). In reality, only 54.2% of the reviews devoted some attention to this macrolevel. Sport scientists and data analysts have emphasized the need for systemic, holistic analyses that consider team sports as a whole entity and explore the interactions between the parts, actively promoting a debate that could redefine training methodology and theory [42–45] and have significant implications for sports pedagogy [46–48].

All levels of analysis potentially provide relevant information, as the whole-part interactions are bidirectional [49]. However, systematic reviews in team sports lack sufficient analyses at the macrolevel of performance. While nine reviews reported on group dynamics and three presented dyadic interactions, the molecular (i.e. individual) level of analysis was clearly predominant, as it was present in 95.8% of the reviews. The excessive focus at the molecular level, coupled with the reduced attention given to contextual factors (as will be explored further in the discussion), creates a bias in analyses of team sports dynamics that excessively favours the parts of the team (subgroups), without considering their interactions.

A dynamic systems approach suggests that there are bidirectional links between two levels (i.e. the macrolevel influences the microlevel and vice versa) [50]. However, some theoretical questions must be addressed before better research designs are implemented. Such questions include the following: (1) “What specific mechanisms mediate the interactions between the micro and macro levels of performance, and how do they vary depending on the stability of the macro level?”; (2) “What mediating effects do the dyadic and group levels have on the interaction between the micro and macro levels?”; (3) “How effective are interventions aiming to improve the interaction between the micro and macro levels?”; and (4) “What effects do such interventions have on the dyadic and group levels of performance?”.

Intermediate levels, such as dyadic and subgroup analyses, have received even less attention. Furthermore, the psychosocial aspects of performance in team sports and the performance behaviours of female participants were largely neglected in the included systematic reviews and meta-analyses. Match context (i.e. match period, location and status, and the quality of the opposition) and its effects on performance are still not well understood, as they are not often considered in previous research.

Future research on team sports should devote increased attention to the macrolevel factors to establish which factors vary and which remain relatively stable across matches. Such research could help to further establish key patterns and fluctuations in behaviour and performance. As mentioned previously, it could be meaningful to analyse the performance of each team separately. If the range of behaviours shown by a team is too narrow, the team might lack adaptability; if the range is too broad, perhaps the team lacks coherence and stability. Coaches could adjust their game models based on these ranges and update the frameworks used to guide the design of practice programmes, thereby more closely aligning players’ intentions with their behaviours. To date, studies focused on the macrolevel have tended to use pooled values (i.e. by aggregating data from several teams; see [51, 52] for exceptions). These two studies [51, 52] focused on the macrolevel while considering interteam variability in gameplay patterns, indicating issues for future studies to address and their applications.

### Dimension of Analysis

Most of the selected reviews integrated more than one dimension of performance in their analyses. Naturally, due to the nature of the research question, some of the reviews exclusively focused on specific topics related to physical or physiological demands [29, 35, 36, 38], tactical dimensions [24, 26, 34], or technical or biomechanical dimensions [23]. Studies that analysed more than one dimension lacked sufficient evidence to establish relationships between dimensions within the same performance event. This topic clearly requires further exploration in future research. Using position-based measures could combine the strengths of notational analyses that traditionally focus on outcomes, while position-based information can provide further information about a collective organization by focusing on the processes conducted until the outcome is reached. For example, position data can enhance the recognition of collective patterns (either in defensive or attacking moments) and characterize team dynamics before critical events that are traditionally classified by notational analysis (e.g. goal-scoring opportunities). The few examples of such combinations included a study that combined tactical network analysis and physical demands to explore the relationships between both [53] and a study that integrated position-data measures with physical demands [54]. Future reviews could focus on establishing links between dimensions based on such examples of integrated analytic approaches.

### Small-Sided Games Versus Formal Matches

Physiological or physical, technical, and tactical measures are often considered when examining training sessions and competitive matches. Across the reviewed studies, small-sided games were the main type of training design considered in match analyses [3, 22–24, 28, 31, 39]. Some reviews on small-sided games exclusively focused on the tactical dimension [3, 24, 31], physical dimension [22], and technical dimension [23]. The two remaining reviews summarized multiple performance dimensions [28, 39].

Studies that have analysed tactical dimensions [3, 24, 31], commonly using a mesolevel (group) analysis, were more common, emphasizing the following types of measures: (1) determining the geometrical centre of the teams; (2) establishing relationships (i.e. distances) between players and the geometrical team centre; and (3) elucidating areas occupied by players as a subgroup. For some of these measures (i.e. those predominantly dependent on positional data), nonlinear analyses (e.g. entropy, relative phase) were used to test the synchronization or regularity of events to identify the dynamic properties of competitive games [3, 24, 31]. In reviews of technical performance dimensions, molecular-level analysis was the most common. Furthermore, notational analysis techniques were the main type used to quantify the frequency of players’ actions and events emerging during games [23].

Finally, studies that analysed the physical or physiological demands of small-sided games [22, 28] used self-report instruments (ratings of perceived exertion) or tracking instruments (e.g. heart rate monitors, global positioning systems) to observe how variations in game formats and playing areas contributed to changes in the physiological intensities and external load demands. Research also investigated how other constraints (e.g. types of playing area markings, use of smaller or larger goals, limitations on ball touches) [28] can lead to adaptations in the variation of training loads; however, such research did not provide robust evidence due to the limitations of the experimental designs used.

The reviews predominantly analysing the physical performance dimensions [25, 29, 35, 36, 38], tended to address highly specific questions. Field et al. [25] reported on the decremental effects of extra time on aspects of players’ physical performance (e.g. distances covered) and their impacts on recovery. Two reviews revealed that physical demands in rugby are closely linked to playing roles [35, 36], revealing methodological inconsistencies in recording high-speed movements that need to be resolved in future research [36]. Additionally, Vieria et al. [29] confirmed the influence of playing roles on physical demands in football, identifying the need to record and assess biochemical measures and the recovery status of participants.

In reviews on tactical and technical performance dimensions, the group and team levels were the main levels of analysis, with game patterns and set pieces or special plays being analysed recurrently [26, 32, 34, 37, 39]. The main outcomes were obtained through notational analyses or observational methodologies. Interestingly, reviews that used more *position-based* measures focused on performance in small-sided games. The two exceptions were the reviews conducted by Sarmento et al. [5] and Rico-González et al. [31], who reported position-based measures in full-sided competitive matches. The integration of position-based measures in competitive matches, combined with the use of nonlinear statistics or data analysis techniques, could become more common in future research and could provide relevant information to complement observational analysis.

### Sociodemographic and Environmental Constraints

Individual sociodemographic constraints such as age, sex, and expertise are used in sports as criteria for the social organization of practices and competitive matches. Most reviews imposed no restrictions on participants’ age or expertise level. However, nine of them included restrictions on age, seven of which focused only on adults [21, 25, 32, 34, 35, 37, 38] and two of which focused solely on young players [29, 39]. Some of the reviews emphasized the pertinence of age or expertise level when presenting or discussing results [3, 23, 24]. For example, Clemente et al. [24] highlighted the relevance of age groups and expertise levels in their observations of specific micro- and mesolevel outcomes related to tactical analyses. They observed that older athletes were more likely to disperse across the field and explore the width of the pitch than younger players, who tended to cluster together. Similarly, in a review of technical performance outcomes, substantial differences were observed in the frequency and accuracy of actions between older and younger players, as well as between athletes with high and low skill levels [23]. Skill-level comparisons were also investigated in a systematic review [3] that revealed variations in specific tactical outcomes at the professional or amateur levels or in skilled and less skilled youth players.

Our results revealed a sex bias in sport sciences research as women’s experiences are misrepresented. This observation is in line with the underrepresentation of women observed in sports and exercise medicine research [55]. Only one review included in this umbrella review focused solely on women [20], and only one more contained a balanced representation of men and women [21]. The other review, including men and women, contained an unbalanced representation, which the authors highlighted as a persistent problem [3]. Thus, only two (~ 8.3%) of the 24 systematic reviews that met our criteria provided relevant information on female players. While some systematic reviews may have been limited by the scope of available investigations, this possibility confirms the problem at the level of planning and conceptualizing original research studies.

Most systematic reviews deliberately aim to solely analyse research on male participants. This is troublesome for two reasons. First, it reflects a predominant social bias that leads to the overrepresentation of male participants while underrepresenting female participants at multiple levels in sports science research [56]. Second, female participants may have different needs, regularities, and demands than male participants. For example, the coping strategies of female athletes differ from those of male athletes, which changes the dynamics of training [57]. Furthermore, performance regularities in several sports have been shown to differ between men and women at similar competitive levels [58–60]. The dominance of male participants in research extends to other fields within sport sciences, such as resistance training [61], underlining how engrained this social bias is.

### Match Context

Contextual or environmental factors constitute one of the core aspects of performance [62]. However, our umbrella review shows that few contextual factors have been considered in existing analyses. Match period was the most commonly considered contextual factor, but even this factor was included in only 45.8% of the included systematic reviews. Thus, despite being recognized as an important variable for understanding game patterns—as teams strategically approach different match periods in a distinct way [63]—the match period is still not widely considered in research on performance. The most common conclusion is that most performance indicators decline during later match periods, probably due to fatigue. However, the emergence of greater scoring opportunities during later match periods has been suggested in some sports (e.g. futsal [30] and in handball [32]). Furthermore, in basketball, it has been suggested that losing teams apply more defensive pressure in the last two periods of a match [18]. Moreover, Silva et al. [37] suggested that the match period is associated with different strategic behaviours in the serving and attacking actions among volleyball players.

Match location, quality of opposition, and match status have also been found to strongly impact sports performance [64–66]. Notwithstanding, the present umbrella review revealed that only five systematic reviews considered match location. As previously mentioned, 45.8% of the systematic reviews made no mention of any of the abovementioned contextual factors. According to some studies, match location is an important variable in handball and football, as teams have a statistically higher likelihood of winning home matches than away matches and exhibit improved performance indicators during home matches [5, 27, 32]. However, most of the studies included in each review did not clearly examine this variable (e.g. Colomer et al. [34], Courel-Ibáñez et al. [18]).

Only four reviews included information on match status. Silva et al.’s [37] review contains only one study in volleyball that focused on this variable, which suggested that it interferes with strategic options; however, a detailed explanation was not provided. Meanwhile, Sarmento et al. [5] suggested that balanced and unbalanced match scenarios were associated with distinct tactical behaviours in football, but the data were sparse and heterogeneous. Overall, the effects of match status effects on the tactical behaviours of teams cannot be generalized.

Finally, three reviews examined the quality of opposition. However, the results of these studies [27] were difficult to compare due to inadequate reporting and inconsistent definitions. Moreover, most of the studies included in another review did not mention the quality of opposition, and detailed information is not presented in the few studies that did address this topic [34]. Finally, Silva et al.’s [37] review included only one study that analysed the quality of opposition; however, the information provided in this study is generic, simply stating that there are differences in gameplay. Thus, information on the quality of opposition is scarce, and no generalizations can be made currently.

Moreover, the impact of social environments (e.g. intrateam relationships, fan support, broadcasting) has not been properly analysed. For example, the 2020–2021 COVID-19 pandemic has created novel challenges in sports that have resumed competition, such as football. It has been suggested that the absence of spectators has changed teams’ performance and that TV broadcasting does not replace live audiences [67]. Furthermore, interactions on social media have changed the public perception of specific players or teams, which, in turn, can change the relationship between the public and a performer during official competitions [68].

Overall, a symbiotic relationship is suggested to exist between spectators and performers [69], implying the need to investigate how attendant spectators and broadcasting influence how performers approach competitions. Additionally, performance indicators are pursued through concrete interpersonal relationships, whereby sociometric data are relevant for understanding how individuals on a team perform when faced with particular task and environmental constraints [70]. It would be interesting to investigate which factors impact athletes’ performance, both quantitatively and qualitatively.

### Limitations

A possible limitation of this systematic review is that it only includes studies published in English in the Web of Science, PubMed, Cochrane Library, Scopus, and SPORTDiscus databases; thus, relevant publications in other languages (and grey literature) could have been overlooked. Additionally, the inclusion of all of the systematic reviews, regardless of the results of their methodological quality (as well as the weak/moderate interrater agreement in some items), can be considered a limitation as some flaws in critical domains can affect the validity of a review and its conclusions. Of note, the items considered “critical domains” can significantly influence the final overall quality rating of a review.

## Future Recommendations

Our results highlight a scarcity of meta-analyses on team sports performance, and a lack of a well-defined population, intervention, comparison, outcome, and study design (PICOS). Future systematic reviews on this topic should preregister their protocols, and more than one author should select and extract the data. Moreover, evaluations of the RoB of included articles should be mandatory, and reviews should be clearer about what type of study design is acceptable for eligibility. For example, the results of single-group, interventional studies should not be compared with those of randomized controlled trials. Additionally, most studies tended to use cross-sectional designs, which creates a need for more longitudinal studies, especially those using randomized controlled designs. Additionally, this bias signifies that so-called predictive analyses are not truly predictive, as cross-sectional designs have been used. In a sense, these analyses are not truly predictive but are “postdictive”.

Although the included studies focused on team sports, individual-level analyses were conducted in 23 of the 24 reviews, while the team level was analysed in slightly more than half of the studies. The interactions between these two levels are currently unclear and need further theoretical interpretation because the interactions between the macro- and microlevels are not entirely clear.

The inductive analysis of large databases has been an increasingly common practice in the era of big data. However, inductive studies capturing hidden patterns (e.g. sequential analysis, t-pattern analysis) to sequence emergent game actions remain scarce. Detecting “stable” patterns of play (i.e. patterns that can be repeated, in some way, in future performances) is difficult but crucial to match analyses. Nevertheless, analyses of specific game events (viewed as perturbations) that cause imbalances in an opposing team are one of the paths that can differentiate the scope of match analysis and can benefit from theoretical guidance. Examples of related questions include: (1) “Are perturbations more strongly influenced by a specific level of performance (e.g., macro-level versus dyadic-level)?”; (2) “Are these perturbations specific to certain game moments or scenarios, i.e., are the effects of such perturbations nonlinear)?”; (3) “Do perturbations of the opposition also perturb the performance of a team (e.g., could microlevel behaviours perturb both teams)?”; (4) “To what extent do these perturbations affect the remainder of a match?”; and (5) “In this vein, should match analysis studies separate their analyses into *pre- and postperturbation* moments?”. These perturbations can result from the actions of the player in possession of the ball, or they can result from the actions of players who might not directly contact the ball. Defining observational tools and procedures that allow researchers to understand how these moments (which often determine the outcome of a game) arise is one of the most attractive challenges to be addressed by future performance analysis.

Despite the large between-match variability of measures (technical, tactical, and physical parameters), some variability can be replicated experimentally. For example, interventions could use the following strategies to improve match analysis in practice contexts: (1) standardize game formats and only compare the same formats (i.e. same number of players, space, constraints, and rules); (2) compare similar training days to avoid variability induced by different training session sequences and contents (e.g. data obtained on match day + 1 day would be compared only with other data obtained on match day + 1 day); (3) base assessments could be made based on ranges instead of single values (e.g. confidence interval or the standard deviation values could be considered instead of mean values), and close attention could be paid to how that range evolves over time; (4) before pretests, proper familiarization with all game formats and tests could be provided to prevent comparisons between pretest and posttest data from being confounded by adaptation to the tests; and (5) all tests have inherent measurement errors—if such errors are calculated (e.g. CVs, ICCs, TEMs), they could provide a means for distinguishing error-related variability from functional variability. By taking these steps, researchers and practitioners could better assess stable changes in task performance instead of random fluctuations or different task-induced variations.

Furthermore, performance analysis conducted in competitive matches could closely examine the role of variable ranges, as greater variability would be expected in such cases. Moreover, analysing the performance of specific teams, instead of aggregating data from different teams, could provide a more accurate overview of how team games unfold. In some sports, match difficulty could be considered while comparing matches of similar expected difficulty levels. Matches could be compared a posteriori based on how balanced the final score was. Although these items will not provide an understanding of all sources of variability, they will provide a better understanding than averaged values.

The involvement of coaches and performance analysts in this process (for example, through conducting interviews) could be fruitful as these professionals will be positioned to decipher specific data that sometimes “occlude” the main information relevant to decision-making [71]. In this way, future match analyses should also complement big data analyses with small data analyses, thus benefiting from the theoretical frameworks developed in psychology for longer than a century [9].

This challenge is significant for researchers who need to align all these new technologies and techniques to carry out data analysis according to the needs of coaching and support staff. An additional part of this challenge is creating effective relationships between all practitioners to produce relevant information that can improve performance by continuously adapting the training designs. Moreover, a holistic theoretical framework that matches analysis in team sports must be implemented. Such a framework should consider a set of relevant variables (e.g. physiological, physical, psychological, environmental) that can be effectively analysed (preferably in real time) to provide technical support staff with coherent and meaningful knowledge [5].

## Conclusion

Research on match analysis in team ball sports has been the subject of growing interest over the past 10 years. However, a considerable number (77% in this present umbrella review) of systematic reviews and meta-analyses have been published in the past two years (i.e. 2019 and 2020). In addition to the potential weaknesses (e.g. small sample sizes, lack of operational definitions) identified in the original studies, our review of the systematic reviews and meta-analysis displayed an overall critically low methodological quality, revealing the need for significant improvements in methodological procedures in such reviews.

Despite focusing on collective sports, player-level analyses of team performance remain dominant in the reviewed studies. Additionally, more research is needed on dyadic, group, and team-level interactions in performance. Finally, existing studies must improve the capacity for combining different dimensions of analysis (i.e. physical, technical, tactical, and psychosocial) to better explain performance in team ball sports and bridge the gap between theory and practice.

## Supplementary Information

**Additional file 1: Table S1**. Interrater reliability values of AMSTAR 2 items and references for studies included in the analyses

## Data Availability

Not applicable.

## References

[1] Evers J, Fullerton H (1910). Touching second; the science of baseball.

[2] Sarmento H, Marcelino R, Anguera M, Campaniço J, Matos N, Leitão J (2014). Match analysis in football: a systematic review. J Sports Sci.

[3] Low B, Coutinho D, Gonçalves B, Rein R, Memmert D, Sampaio J (2020). A systematic review of collective tactical behaviours in football using positional data. Sports Med.

[4] González-Víllora S, Serra-Olivares J, Pastor-Vicedo JC, da Costa IT (2015). Review of the tactical evaluation tools for youth players, assessing the tactics in team sports: football. Springerplus.

[5] Sarmento H, Clemente FM, Araújo D, Davids K, McRobert A, Figueiredo A (2018). What performance analysts need to know about research trends in association football (2012–2016): a systematic review. Sports Med.

[6] Araújo D, Bourbousson J, Passos P, Davids K, Chow J (2016). Theoretical perspectives on interpersonal coordination for team behavior. Interpersonal coordination and performance in social systems.

[7] Araújo D, Davids K (2016). Team dynergies in sport: theory and measures. Front Psychol.

[8] Araújo D, Passos P, Esteves P, Duarte R, Lopes J, Hristovski R (2015). The micro-macro link in understanding sport tactical behaviours: integrating information and action at different levels of system analysis in sport. Mov Sport Sci/Sci Mot.

[9] Araujo D, Micael C, Seifert L, Sarmento H, Davids K (2021). Artificial Intelligence in Sport Performance Analysis.

[10] Woo S, Tay L, Proctor W (2020). Big data in psychological research.

[11] Davids K, Araújo D, Shuttleworth R, Reilly J, Cabri J, Araújo D (2005). Applications of dynamical dystems theory to football. Science and football V.

[12] Travassos B, Davids K, Araújo D, Esteves TP (2013). Performance analysis in team sports: advances from an ecological dynamics approach. Int J Perform Anal Sport.

[13] Moher D, Liberati A, Tetzlaff J, Altman DG (2009). Preferred reporting items for systematic reviews and meta-analyses: the PRISMA statement. BMJ.

[14] Page MJ, McKenzie JE, Bossuyt PM, Boutron I, Hoffmann TC, Mulrow CD (2021). The PRISMA 2020 statement: an updated guideline for reporting systematic reviews. BMJ.

[15] Shea BJ, Reeves BC, Wells G, Thuku M, Hamel C, Moran J (2017). AMSTAR 2: a critical appraisal tool for systematic reviews that include randomised or non-randomised studies of healthcare interventions, or both. BMJ.

[16] McHugh ML (2012). Interrater reliability: the kappa statistic. Biochem Med (Zagreb).

[17] Almeida MO, Yamato TP, Parreira P, Costa LOP, Kamper S, Saragiotto BT (2020). Overall confidence in the results of systematic reviews on exercise therapy for chronic low back pain: a cross-sectional analysis using the Assessing the Methodological Quality of Systematic Reviews (AMSTAR) 2 tool. Braz J Phys Ther.

[18] Courel-Ibáñez J, McRobert AP, Toro EO, Vélez DC (2017). Collective behaviour in basketball: a systematic review. Int J Perform Anal Sport.

[19] Maimón AQ, Courel-Ibáñez J, Ruíz FJR (2020). The basketball pass: a systematic review. J Hum Kinet.

[20] Reina M, García-Rubio J, Ibáñez SJ (2020). Training and competition load in female basketball: a systematic review. Int J Environ Res Public Health.

[21] Medeiros AIA, Palao JM, Marcelino R, Mesquita I (2014). Systematic review on sports performance in beach volleyball from match analysis. R Bras Cinean Desemp Hum.

[22] Bujalance-Moreno P, Latorre-Roman PA, Garcia-Pinillos F (2019). A systematic review on small-sided games in football players: acute and chronic adaptations. J Sports Sci.

[23] Clemente FM, Sarmento H (2020). The effects of small-sided soccer games on technical actions and skills: a systematic review. Human Mov.

[24] Clemente FM, Afonso J, Castillo D, Arcos AL, Silva AF, Sarmento H (2020). The effects of small-sided soccer games on tactical behavior and collective dynamics: a systematic review. Chaos Solitons Fractals.

[25] Field A, Naughton RJ, Haines M, Lui S, Corr LD, Russell M (2020). The demands of the extra-time period of soccer: a systematic review. J Sport Health Sci.

[26] Goes FR, Meerhoff LA, Bueno MJO, Rodrigues DM, Moura FA, Brink MS (2020). Unlocking the potential of big data to support tactical performance analysis in professional soccer: a systematic review. Eur J Sport Sci.

[27] Sarmento H, Marcelino R, Anguera MT, CampaniÇo J, Matos N, LeitÃo JC (2014). Match analysis in football: a systematic review. J Sports Sci.

[28] Sarmento H, Clemente FM, Harper LD, Costa IT, Owen A, Figueiredo AJ (2018). Small sided games in soccer—a systematic review. Int J Perform Anal Sport.

[29] Vieira LHP, Carling C, Barbieri FA, Aquino R, Santiago PRP (2019). Match running performance in young soccer players: a systematic review. Sports Med.

[30] Agras H, Ferragut C, Abraldes JA (2016). Match analysis in futsal: a systematic review. Int J Perform Anal Sport.

[31] Rico-González M, Pino-Ortega J, Clemente F, Rojas-Valverde D, Los AA (2021). A systematic review of collective tactical behavior in futsal using positional data. Biol Sport.

[32] Ferrari MR, Sarmento H, Vaz V (2019). Match analysis in handball: a systematic review. Mont J Sports Sci Med.

[33] Ferraz A, Valente-Dos-Santos J, Sarmento H, Duarte-Mendes P, Travassos B (2020). A review of players' characterization and game performance on male rink-hockey. Int J Environ Res Public Health.

[34] Colomer CME, Pyne DB, Mooney M, McKune A, Serpell BG (2020). Performance analysis in rugby union: a critical systematic review. Sports Med Open..

[35] Glassbrook DJ, Doyle TLA, Alderson JA, Fuller JT (2019). The demands of professional rugby league match-play: a meta-analysis. Sports Med Open.

[36] Hausler J, Halaki M, Orr R (2016). application of global positioning system and microsensor technology in competitive rugby league match-play: a systematic review and meta-analysis. Sports Med.

[37] Silva M, Marcelino R, Lacerda D, Joao PV (2016). Match analysis in volleyball: a systematic review. Mont J Sports Sci Med.

[38] Harper DJ, Carling C, Kiely J (2019). High-intensity acceleration and deceleration demands in elite team sports competitive match play: a systematic review and meta-analysis of observational studies. Sports Med.

[39] Fernández-Espínola C, Abad Robles MT, Giménez Fuentes-Guerra FJ (2020). Small-sided games as a methodological resource for team sports teaching: a systematic review. Int J Environ Res Public Health.

[40] Davids K, Araújo D, Vilar L, Renshaw I, Pinder R (2013). An ecological dynamics approach to skill acquisition: implications for development of talent in sport. Talent Dev Excel.

[41] Davids K, Gullich A, Shuttleworth R, Araújo D, Baker J, Cobley S, Schorer J, Wattie N (2017). Understanding environmental and task constraints on talent development. Routledge handbook of talent identification and development in sport.

[42] Pol R, Balague N, Ric A, Torrents C, Kiely J, Hristovski R (2020). Training or synergizing? Complex systems principles change the understanding of sport processes. Sports Med Open.

[43] Glazier PS (2017). Towards a grand unified theory of sports performance. Hum Mov Sci.

[44] Button C, Seifert L, Chow J-Y, Araújo D, Davids K (2020). Dynamics of skill acquisition.

[45] Araújo D, Dicks M, Davids K, Cappuccio ML (2019). Selecting among affordances: A basis for channeling expertise in sport. Handbook of embodied cognition and sport psychology.

[46] Richards P, Collins D, Mascarenhas DRD (2017). Developing team decision-making: a holistic framework integrating both on-field and off-field pedagogical coaching processes. Sports Coach Rev.

[47] Correia V, Carvalho J, Araújo D, Pereira E, Davids K (2019). Principles of nonlinear pedagogy in sport practice. Phys Educ Sport Pedagogy.

[48] Renshaw I, Araújo D, Button C, Chow JY, Davids K, Moy B (2016). Why the constraints-led approach is not teaching games for understanding: a clarification. Phys Educ Sport Pedagogy.

[49] Argyris J, Faust G, Haase M, Friedrich R (2015). An exploration of dynamical systems and chaos. Completely revised and enlarged second edition.

[50] Strogatz S (2015). Nonlinear dynamics and chaos: with applications to physics, biology, chemistry, and engineering.

[51] Laporta L, Medeiros AIA, Vargas N, Castro HO, Bessa C, João PV (2021). coexistence of distinct performance models in high-level women's volleyball. J Hum Kinet.

[52] Martins JB, Mesquita I, Mendes A, Santos L, Afonso J (2021). Inter-team variability in high-level women’s volleyball from the perspective of Social Network Analysis: an analysis in critical game scenarios. Int J Perform Anal Sport.

[53] Castellano J, Echeazarra I (2019). Network-based centrality measures and physical demands in football regarding player position: Is there a connection? A preliminary study. J Sports Sci.

[54] Gonçalves B, Esteves P, Folgado H, Ric A, Torrents C, Sampaio J (2017). Effects of pitch area-restrictions on tactical behavior, physical, and physiological performances in soccer large-sided games. J Strength Cond Res.

[55] Costello JT, Bieuzen F, Bleakley CM (2014). Where are all the female participants in sports and exercise medicine research?. Eur J Sport Sci.

[56] Evans AB, Pfister GU (2020). Women in sports leadership: A systematic narrative review. Int Rev Sociol Sport.

[57] Bojkowski Ł, Kalinowski P, Kalinowska K, Jerszyński D (2020). Coping with stress among women and men training team sports games. J Phys Educ Sport.

[58] Lima R, Palao JM, Moreira M, Clemente FM (2019). Variations of technical actions and efficacy of national teams’ volleyball attackers according to their sex and playing positions. Int J Perform Anal Sport.

[59] Maneiro R, Casal CA, Ardá A, Losada JL (2019). Application of multivariant decision tree technique in high performance football: the female and male corner kick. PLoS ONE.

[60] Vázquez-Diz JA, Morillo-Baro JP, Reigal RE, Morales-Sánchez V, Hernández-Mendo A (2019). Mixed methods in decision-making through polar coordinate technique: differences by gender on beach handball specialist. Front Psychol.

[61] Blazevich AJ, Wilson CJ, Alcaraz PE, Rubio-Arias JA (2020). effects of resistance training movement pattern and velocity on isometric muscular rate of force development: a systematic review with meta-analysis and meta-regression. Sports Med.

[62] Kelso JAS, Putnam CA, Goodman D (1983). On the space-time structure of human interlimb co-ordination. Q J Exp Psych.

[63] Marcelino RO, Sampaio JE, Mesquita IM (2012). Attack and serve performances according to the match period and quality of opposition in elite volleyball matches. J Strength Cond Res.

[64] Aquino R, Munhoz Martins GH, Palucci Vieira LH, Menezes RP (2017). Influence of match location, quality of opponents, and match status on movement patterns in brazilian professional football players. J Strength Cond Res.

[65] Lago C (2009). The influence of match location, quality of opposition, and match status on possession strategies in professional association football. J Sports Sci.

[66] Taylor JB, Mellalieu SD, James N, Shearer DA (2008). The influence of match location, quality of opposition, and match status on technical performance in professional association football. J Sports Sci.

[67] Horky T (2020). No sports, no spectators—no media, no money? The importance of spectators and broadcasting for professional sports during COVID-19. Soccer Soc.

[68] Chmait N, Westerbeek H, Eime R, Robertson S, Sellitto C, Reid M (2020). Tennis influencers: the player effect on social media engagement and demand for tournament attendance. Telemat Inform.

[69] Kim D, Kim A, Kim J, Ko YJ. Symbiotic relationship between sport media consumption and spectatorship: the role of flow experience and hedonic need fulfillment. J Global Sport Man. 2019; 1–23.

[70] Safina DM, Podgornaya AI, Grudina SI, Datsyk AA. Key performance indicators influence to team building. J Social Sci Res. 2018;Special Issue(5):161–5.

[71] Rothwell M, Davids K, Stone JA, O’Sullivan M, Vaughan J, Newcombe D (2020). A department of methodology can coordinate transdisciplinary sport science support. J Expert.

