# Supplementary material for: Match Analysis in Team Ball Sports: An Umbrella Review of Systematic Reviews and Meta-Analyses
Source: Sports Med Open. 2022 May 13;8:66. doi: 10.1186/s40798-022-00454-7 (PMC9100301; doi:10.1186/s40798-022-00454-7)
Supplement: Supplementary file 1 — Additional file 1: Table S1. Interrater reliability values of AMSTAR 2 items and references for studies included in the analyses [file 40798_2022_454_MOESM1_ESM.docx]

Electronic Supplementary Material Table S1 - Interrater reliability values of AMSTAR 2 items and references for studies included in the analyses

| Item | Cohen  Kappa | 95% confidence interval |
| --- | --- | --- |
| 1 | 1.00 | 1.00-1.00 |
| 2 | 1.00 | 1.00-1.00 |
| 3 | 0.70 | 0.32-1.00 |
| 4 | 0.57 | 0.22-0.92 |
| 5 | 0.81 | 0.57-1.06 |
| 6 | 0.92 | 0.75-1.07 |
| 7 | 0.64 | 0.33-0.94 |
| 8 | 0.65 | 0.28-1.00 |
| 9 | 0.53 | 0.25-0.81 |
| 10 | 1.00 | 1.00-1.00 |
| 11 | 1.00 | 1.00-1.00 |
| 12 | 1.00 | 1.00-1.00 |
| 13 | 0.82 | 0.59-1.06 |
| 14 | 0.75 | 0.43-1.07 |
| 15 | 1.00 | 1.00-1.00 |
| 16 | 0.83 | 0.52-1.14 |
